# Evaluation of Serum Zinc Status in Patients With Neurologic Impairment Under Controlled Enteral Nutrition

**DOI:** 10.7759/cureus.98266

**Published:** 2025-12-01

**Authors:** Naoki Hashizume, Hirotomo Nakahara, Takato Aikoh, Hiroki Yoshida, Tomohiro Kurahachi, Saki Sakamoto, Daisuke Masui, Yoshinori Koga, Koshiro Sugita, Tatsuru Kaji

**Affiliations:** 1 Pediatric Surgery, Kurume University School of Medicine, Kurume, JPN; 2 General and Thoracic Surgery, Translational Medicine Program, The Hospital for Sick Children, Toronto, CAN; 3 Pediatric Surgery, Kagoshima University, Kagoshima, JPN

**Keywords:** albumin (alb), enteral nutrition (en), neurologic impairment, serum zinc level, zinc (zn)

## Abstract

Background: Patients with neurologic impairment (NI) often require long-term enteral nutrition. Zinc plays a vital role in neurological and immune function, yet data on zinc intake and status in NI patients remain limited. This study aimed to evaluate whether serum zinc concentrations reflect standardized daily zinc intake in patients with NI receiving fixed-content enteral formulas (EFs) and to identify factors associated with serum zinc levels.

Methods: This retrospective study included bedridden NI patients aged ≥16 years who received EFs via nasogastric or gastrostomy feeding. Clinical parameters, serum biochemical markers, and daily nutrient intake were assessed. Patients were stratified by serum zinc levels (<60 μg/dL or ≥60 μg/dL). Univariate and multivariate linear regression analyses were performed to identify factors independently associated with serum zinc concentrations.

Results: Of 31 patients analyzed, 8 (25.8%) exhibited serum zinc deficiency. Zinc intake was significantly lower in the low zinc group compared to the normal zinc group (9.2 vs. 13.5 mg/day, p = 0.0157). Notably, zinc deficiency was observed even in individuals whose zinc intake exceeded the Estimated Average Requirement (EAR) or Recommended Dietary Allowance (RDA). In regression analyses, both daily zinc intake (β = 1.28, p = 0.0273) and serum albumin (β = 14.87, p = 0.0407) were independently associated with serum zinc levels.

Conclusions: Despite controlled enteral nutrition, a substantial proportion of patients with NI exhibited zinc deficiency. Although limited by a small sample size, these findings highlight the importance of individualized monitoring and potential supplementation beyond standardized intake levels.

## Introduction

Patients with neurologic impairment (NI) often suffer from feeding difficulties and limitations. Several patients require a nasogastric tube or gastrostomy for enteral nutrition and oral pharmacotherapy. NI has been recognized as a critical disorder that requires intensive nutritional support due to the presence of neurological and/or metabolic disorders[1]. Enteral formulas (EFs) are multi-nutrient products, including ready-to-use liquids or puddings, and are recommended for long-term exclusive use in patients with NI who require nasogastric or gastrostomy feeding. The daily intake of EFs in patients with NI can be accurately quantified [2].

Previous reports have indicated that daily zinc intake may be insufficient to meet recommended dietary requirements in specific populations, including children [3], the elderly [4], young women adhering to weight-reduction diets [5], and other at-risk groups. Patients with NI are at high risk for epilepsy, and zinc deficiency has been identified as a contributing factor to increased seizure frequency in this population [6]. Therefore, maintaining adequate zinc levels is essential for the neurological stability of patients with NI. The evaluation of zinc intake is a critical component in the analysis of factors contributing to zinc deficiency. Nevertheless, the accurate assessment of dietary zinc intake remains challenging, and consequently, reports that quantify actual intake are limited, because it is difficult to create a situation in which subjects consume meals with precisely defined nutrient contents.

In the present study, we aimed to determine whether serum zinc concentrations reflect standardized zinc intake and characteristics in patients with NI receiving EFs with a fixed zinc content, in comparison with the reference values established in the Dietary Reference Intakes for Japanese (2025) [7].

## Materials and methods

Patients

This was a single-center retrospective study. Patients with NI aged 16 years or older who were bedridden and required enteral nutrition via a nasogastric tube or gastrostomy at Kurume University Hospital were included between June 2013 and April 2020. C-reactive protein (CRP) levels were below the detectable limit (<0.03 mg/dL) in all patients. Patients with decompensated heart, lung, kidney, or liver failure, or with involuntary weight loss or gain exceeding 5% within the previous three months, were excluded from the study.

Data collection

All the patients underwent a baseline assessment, including age, sex, height, weight, and body mass index (BMI) (calculated as the weight (kg) divided by the height (m) squared, kg/m^2^). Information on carbamazepine use was recorded, and serum biochemical parameters were measured, including total protein, albumin, transferrin, alkaline phosphatase, zinc, and copper. Serum biochemical measurements were performed using an automated chemistry analyzer (LABOSPECT 008α, Hitachi High-Technologies Corporation, Tokyo, Japan).

Serum total protein, albumin, transferrin, and alkaline phosphatase concentrations were determined by the bromocresol purple (BCP) method using the L-Type Wako Albumin BCP reagent (Identification No. 2; improved BCP method; Fujifilm Wako Pure Chemical Corporation, Osaka, Japan). Serum zinc concentration was measured with the Acurus Auto Zn reagent (Shino-Test Corporation, Tokyo, Japan), and serum copper concentration was determined using the Quick Auto Neo Cu reagent (Shino-Test Corporation, Tokyo, Japan). Blood samples were obtained in the morning after an overnight fast, and serum was promptly separated and analyzed to ensure consistency and prevent pre-analytical changes. Nutritional intake was calculated from the actual daily volumes of EFs administered to each patient. Serum zinc levels were assessed according to the 2018 Clinical Practice Guidelines for Zinc Deficiency [8], which define zinc deficiency as a serum zinc concentration of less than 60 μg/dL. The estimated average requirement (EAR) and recommended dietary allowance (RDA) for zinc intake were based on the Dietary Reference Intakes for Japanese (2025), published by the Ministry of Health, Labour and Welfare, Japan [7].

This study was performed after obtaining informed consent from the parents. IRB approval was granted by the Kurume University School of Medicine (Approval No. 17302).

First, patients were divided into two groups based on their serum zinc levels: the low serum zinc group (Zn < 60 μg/dL) and the normal serum zinc group (Zn ≥ 60 μg/dL). A comparative analysis was then conducted between the two groups. Subsequently, to identify factors associated with serum zinc levels, such as age, sex, serum albumin, serum copper level, zinc intake, and carbamazepine use, correlation and multivariate regression analyses were performed using relevant clinical and nutritional parameters.

Statistical analyses

All data are expressed as the median (interquartile range). Comparisons between the low serum zinc group and the normal serum zinc group were performed using the chi-square test for categorical variables and the Mann-Whitney U test for continuous variables. Variables with statistical significance in univariate analyses were subsequently entered into a multivariate linear regression model to identify independent predictors of serum zinc levels. Correlations between continuous variables in both univariate and multivariate analyses were evaluated using Spearman’s rank correlation coefficient test, as the data were not normally distributed. Collinearity among independent variables was assessed using the variance inflation factor (VIF), and variables with VIF values greater than 10 were excluded from the multivariate model. All statistical analyses were performed using the JMP software package (SAS, Cary, NC, USA), and p-values of <0.05 were considered statistically significant.

## Results

A total of 59 patients were identified during the study period. Twenty-eight patients were excluded due to meeting the exclusion criteria and the inability to calculate nutrient intake or assess serum zinc levels. Thirty-one patients (15 males and 16 females) with NI underwent measurements of their intake EFs. The median age was 24 (18.0-35.0) years old. Regarding the causal disorders of NI, 2 patients had a genetic anomaly, 22 had suffered cerebral damage in the neonatal period, and 7 had suffered cerebral damage in infancy or later. None of the participants exhibited clinical symptoms of zinc deficiency, such as dermatological manifestations or glossitis. The hematological findings of the study participants are summarized in Table 1.

**Table 1:** Characteristics, serum biochemical parameters, and daily nutritional intakes of patients with neurological impairment EAR: Estimated Average Requirement, RDA: Recommended Dietary Allowance. ^※^Data are expressed as median (interquartile range). ^※※^Data are expressed as number (%).

	All (n = 31)
Characteristics	
Age (y)^※^	24.0 (18.0, 35.0)
Male (n)^※※^	15 (48.4)
Height (m)^※^	137 (130, 148)
Body weight (kg)^※^	28.2 (21.8, 33)
Body mass index (kg/m^2^)^※^	14.6 (12.4, 17.3)
Serum biochemical parameters^※^	
Total protein (g/dL)	7.28 (6.87, 7.49)
Albumin (g/dL)	3.95 (3.7, 4.2)
Transferrin (mg/dL)	24.1 (20.5, 29.1)
Alkaline phosphatase (U/L)	260 (208, 347)
Zinc (μg/dL)	67 (57, 78)
Cupper (μg/dL)	112 (89, 127)
Carbamazepine (n)^※※^	7 (22.6)
Daily intake	
Energy (kcal/day)^※^	937 (800, 1200)
Energy per kilogram of body weight (kcal/kg/day)^※^	34.51 (29.85, 42.86)
Zinc (mg/day)^※^	12 (9.6, 15)
Zinc per kilogram of body weight (mg/kg/day)^※^	0.41 (0.29, 0.67)
Under EAR zinc intake (n)^※※^	3 (9.7)
Under RDA zinc intake (n)^※※^	4 (12.9)

**Figure 1:**
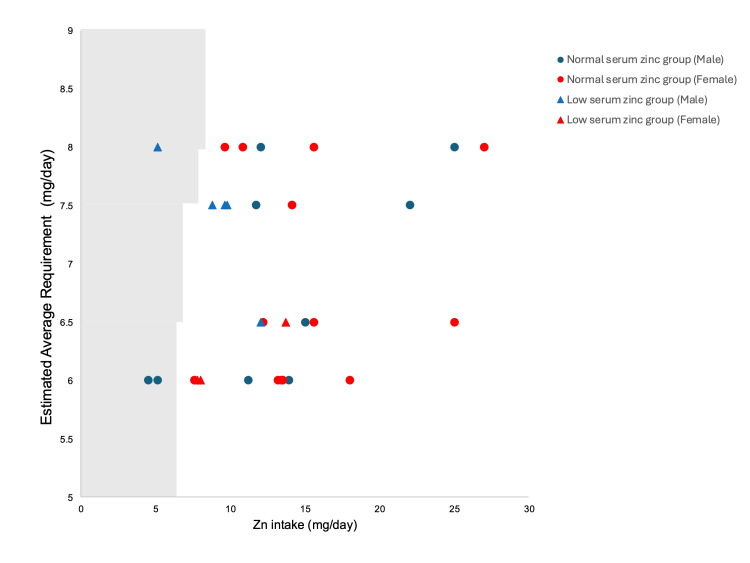
Plot of daily zinc intake and Estimated Average Requirement values in patients with neurologic impairment The Estimated Average Requirement values are based on the Dietary Reference Intakes for Japanese (2025). The points to the left of the line (the shaded area) represent the cases where the administered dose fell below the value.

**Figure 2:**
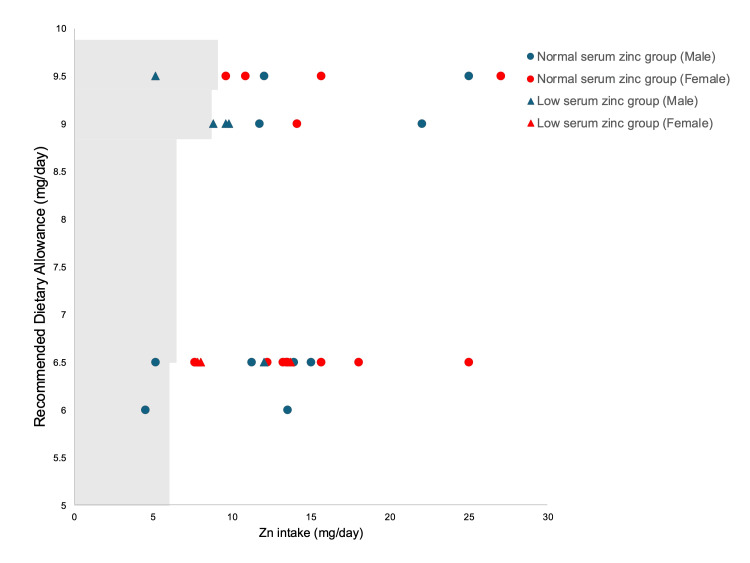
Plot of daily zinc intake and Recommended Dietary Allowance values in patients with neurologic impairment The Recommended Dietary Allowance values are based on the Dietary Reference Intakes for Japanese (2025). The points to the left of the line (the shaded area) represent the cases where the administered dose fell below the value.

The median serum total protein level was 7.28 (6.87-7.49) g/dL, and the median albumin level was 3.95 (3.7-4.2) g/dL. The median transferrin concentration was 24.1 (20.5-29.1) mg/dL. Based on these values, no patients exhibited findings suggestive of malnutrition. Table 1 shows the daily nutritional energy intakes and zinc intakes of patients with NI. The median energy intake was 937 (800, 1200) kcal/day, corresponding to 34.51 (29.85, 42.86) kcal/kg/day. The median zinc intake was 12 (9.6, 15) mg/day, equivalent to 0.41 (0.29, 0.67) mg/kg/day. Among the patients analyzed, three male patients (9.7%) had zinc intakes below the EAR (Figure 1), and four (12.9%) had zinc intakes below the RDA (Figure 2).

Among those who received zinc doses below the EAR or RDA, two patients (6.5%) maintained normal serum zinc concentrations. Conversely, seven patients (22.6%) exhibited low serum zinc levels despite zinc intake above the EAR, and six patients (19.4%) had low serum zinc levels despite zinc intake above the RDA.

Among the 31 patients included in the analysis, 8 were found to have zinc deficiency. Table 2 shows the comparison between the low serum zinc level group (n = 8) and the normal serum zinc level group (n = 23). No significant differences were observed in baseline characteristics, including age, sex, height, body weight, or BMI, between the two groups. Among the serum biochemical parameters, serum zinc levels were significantly lower in the low serum zinc group compared to the normal group (54 (53, 57) vs. 69 (66, 83) U/L; p < 0.0001). Other parameters such as total protein, albumin, transferrin, and copper did not show significant differences. The proportion of patients using carbamazepine was higher in the low serum zinc group (4/8 patients, 50.0%) than in the normal zinc group (3/23 patients, 13.4%) (p = 0.0528), indicating a trend toward significance.

In terms of daily nutritional intake, zinc intake was significantly lower in the low serum zinc group than in the normal group (9.2 (7.76, 11.4) vs. 13.5 (11.25, 15.6) mg/day; p = 0.0157). Similarly, zinc intake per kilogram of body weight was significantly lower in the low zinc group (0.30 (0.27, 0.38) vs. 0.48 (0.36, 0.71) mg/kg/day; p = 0.0145). No significant differences were found in total energy intake or energy intake per kilogram of body weight. There were no significant differences between the two groups in the proportion of patients whose zinc intake was below the EAR or the RDA (Table 2).

**Table 2:** Comparison between the low serum and the normal serum zinc level groups ERA: Estimated Average Requirement, RDA: Recommended Dietary Allowance. ^※^Data are expressed as median (Interquartile range). ※※Data are expressed as number (%). Comparisons between the low serum and the normal serum zinc groups were performed using the chi-square test for categorical variables and the Mann-Whitney U test for continuous variables. p-values less than 0.05 were considered statistically significant.

	Low serum zinc group (n = 8)	Normal serum zinc group (n = 23)	p-value
Characteristics			
Age (y)※	22 (19.25, 40.75)	24 (17, 35)	0.6187
Male (n)※※	3 (37.5)	13 (56.5)	0.6679
Height (m)※	138.5 (133.5, 148.5)	137 (130, 148)	0.6842
Body weight (kg)※	28.6 (26.03, 38.38)	28.2 (21.2, 33)	0.4425
Body mass index (kg/m2)※	14.6 (13.08, 16.93)	14.2 (11.9, 17.9)	0.6034
Serum biochemical parameters※			
Total protein (g/dL)	7.11 (6.91, 7.25)	7.38 (6.85, 7.76)	0.1248
Albumin (g/dL)	3.72 (3.42, 4.03)	3.96 (3.80, 4.33)	0.0993
Transferrin (mg/dL)	22.15 (19.53, 29.03)	26.7 (21.1, 29.1)	0.3547
Alkaline phosphatase (U/L)	288.5 (211.75, 386.75)	238 (191, 319)	0.6679
Zinc (μg/dL)	54 (53, 57)	69 (66, 83)	<0.0001
Cupper (μg/dL)	109.5 (89.25, 123)	112 (89, 133)	0.484
Carbamazepine (n)※※	4 (50.0)	3 (13.4)	0.0528
Daily intake			
Energy (kcal/day)※	950 (800, 1237.5)	937 (900, 1200)	1
Energy per kilogram of body weight (kcal/kg/day)※	30.19 (20.62, 46.74)	34.72 (30.82, 42.45)	0.4563
Zinc (mg/day)※	9.2 (7.76, 11.4)	13.5 (11.25, 15.6)	0.0157
Zinc per kilogram of body weight (mg/kg/day)※	0.30 (0.27, 0.38)	0.48 (0.36, 0.71)	0.0145
Under EAR zinc intake (n)※※	1 (12.5)	2 (8.7)	0.2638
Under RDA zinc intake (n)※※	2 (25.0)	2 (8.7)	1

Factors associated with serum zinc levels were evaluated using univariate and multivariate linear regression analyses (Table 3).

**Table 3:** Univariate and multivariate linear regression analyses for serum zinc levels

	Univariate analysis			Multivariate analysis		
Variable	Standardized β (95% CI)	t-value	p-value	Standardized β (95% CI)	t-value	p-value
Albumin (g/dL)	15.75 (0.56, 30.94)	2.10	0.0426	14.87 (0.68, 29.06)	2.12	0.0407
Copper level (µg/dL)	0.16 (-0.09, 0.41)	1.28	0.2076			
Zinc intake (mg/day)	1.35 (0.15, 2.54)	2.30	0.0282	1.28 (0.15, 2.42)	2.33	0.0273
Carbamazepine	4.70 (-3.74, 13.13)	1.12	0.2638			
Age (years)	0.02 (-0.68, 0.73)	0.06	0.9494			
Sex (male)	1.66 (-5.53, 8.85)	0.47	0.6400			

In the univariate analysis, serum zinc level was significantly associated with albumin concentration (β = 15.75, 95% CI: 0.56-30.94, p = 0.0426) and daily zinc intake (β = 1.35, 95% CI: 0.15-2.54, p = 0.0282).

No significant associations were observed for copper (β = 0.16, 95% CI: -0.09 to 0.41, p = 0.2076), carbamazepine use (β = 4.70, 95% CI: -3.74 to 13.13, p = 0.2638), age (β = 0.02, 95% CI: -0.68 to 0.73, p = 0.9494), or sex (male) (β = 1.66, 95% CI: -5.53 to 8.85, p = 0.6400). In the multivariate analysis, albumin (β = 14.87, 95% CI: 0.68-29.06, p = 0.0407) and zinc intake (β = 1.28, 95% CI: 0.15-2.42, p = 0.0273) remained independently associated with serum zinc levels.

## Discussion

This study demonstrated the following key findings: (1) zinc deficiency was observed even in individuals whose zinc intake exceeded the RDA, such as the EAR and RDA outlined in the Dietary Reference Intakes among patients with NI receiving EFs; (2) there was a significant association between serum zinc concentrations and daily zinc intake; and (3) serum albumin was also found to be an independent predictor of serum zinc concentration.

Previous studies have questioned the reliability of dietary intake assessments for estimating serum trace element levels because of differences in bioavailability and gastrointestinal absorption [9]. Interestingly, serum zinc deficiency was observed even in patients whose zinc intake exceeded the EAR or RDA. Our results indicate that actual zinc intake significantly affects serum zinc status, and insufficient intake contributes to deficiency. However, even among patients receiving enteral nutrition with a standardized composition, individual variability in zinc absorption and metabolism may influence serum levels. Factors such as gastrointestinal function, inflammation, albumin concentration, and drug interactions can alter zinc bioavailability and distribution. In this study, several male patients received zinc doses below the EAR and RDA because these reference values are set higher for males than for females of the same age group. Moreover, patients with neurological impairment generally have lower body weight and therefore receive smaller absolute amounts of energy and zinc. These findings suggest that zinc supplementation should be considered for such patients. Conversely, some individuals exhibited low serum zinc concentrations despite intakes above the EAR or RDA, indicating that serum zinc levels are affected by multiple factors, including serum albumin, and underscoring the need for regular monitoring.

Serum albumin was identified as an independent predictor of serum zinc concentration in this study, consistent with the findings of Ruangritchankul et al. [10]. Although no patients in our cohort exhibited severe hypoalbuminemia, those with neurological impairment and low albumin levels may be at increased risk of zinc deficiency. Because hypoalbuminemia often reflects malnutrition or inflammation [9], both known to reduce serum zinc, maintaining adequate nutritional status and controlling inflammation are essential to prevent deficiency. While a significant correlation between zinc intake and serum levels was observed, serum zinc concentration is also influenced by homeostatic regulation, acute-phase responses, and protein-binding dynamics. Therefore, assessing both dietary intake and biochemical parameters is necessary for a comprehensive evaluation of zinc status.

Although carbamazepine use was not significantly associated with serum zinc levels, a higher proportion of patients in the low zinc group were receiving this medication, suggesting a possible trend. Previous studies have proposed that long-term carbamazepine therapy may alter trace element metabolism and contribute to zinc deficiency, though the evidence remains inconclusive [11]. The drug is thought to induce hepatic microsomal enzymes and enhance the metabolism or excretion of trace elements such as zinc and selenium, or to affect intestinal absorption [11]. Conversely, other reports have found no consistent relationship between carbamazepine use and serum zinc levels, particularly among patients with stable nutrition and chronic administration [12,13]. In any case, serum zinc should be monitored during carbamazepine therapy, as zinc contributes to neuronal stability and antioxidant defense, and its deficiency may increase neuronal excitability and seizure susceptibility during febrile episodes.

Although no overt clinical signs of zinc deficiency were observed, 25.8% of patients were biochemically deficient (Table 2). This underscores the need for proactive monitoring, especially in neurologically impaired individuals, where zinc status may affect seizure control and immune function.

This study has several limitations. First, it was a retrospective, single-center study with a limited sample size. Second, potential confounding factors such as gastrointestinal losses and medication-induced alterations in trace element metabolism were not assessed. In addition, serum zinc levels may fluctuate with acute-phase responses and circadian variation. Future studies should adopt longitudinal or interventional designs and include comprehensive markers of zinc homeostasis, such as metallothionein expression or zinc transporter activity. Expanding the scope to include dietary interventions may also help define optimal zinc requirements in patients with neurological impairment. As a future perspective, prospective studies with larger sample sizes should be considered to confirm these results.

## Conclusions

Serum zinc levels showed a weak correlation with the administered zinc intake. Notably, some patients exhibited low serum zinc levels despite meeting the reference intake values set by the Dietary Reference Intakes for Japanese. Since patients with NI receive EFs under caregiver and medical supervision, regular monitoring of zinc status and appropriate supplementation should be necessary.

## References

[1] Diamanti A, Capriati T, Mosca A (2023). Neurological impairment and malnutrition in children: the role of home enteral nutrition in real life. Front Nutr.

[2] Orel A, Homan M, Blagus R, Benedik E, Orel R, Fidler Mis N (2018). Nutrition of patients with severe neurologic impairment. Radiol Oncol.

[3] Vreugdenhil M, Akkermans MD, van der Merwe LF, van Elburg RM, van Goudoever JB, Brus F (2021). Prevalence of zinc deficiency in healthy 1-3-year-old children from three Western European countries. Nutrients.

[4] Mocchegiani E, Romeo J, Malavolta M, Costarelli L, Giacconi R, Diaz LE, Marcos A (2013). Zinc: dietary intake and impact of supplementation on immune function in elderly. Age (Dordr).

[5] Cheng HL, Griffin HJ, Bryant CE, Rooney KB, Steinbeck KS, O'Connor HT (2013). Impact of diet and weight loss on iron and zinc status in overweight and obese young women. Asia Pac J Clin Nutr.

[6] Seven M, Basaran SY, Cengiz M, Unal S, Yuksel A (2013). Deficiency of selenium and zinc as a causative factor for idiopathic intractable epilepsy. Epilepsy Res.

[7] (2025). Ministry of Health Labour and Welfare. Dietary reference intakes for Japanese people. https://www.mhlw.go.jp/stf/seisakunitsuite/bunya/kenkou_iryou/kenkou/eiyou/syokuji_kijyun.html.

[8] Kodama H, Itakura H, Omori H (2018). Practice guideline for zinc deficiency. Jpn Soc Clin Nutr..

[9] Obara H, Tomite Y, Doi M (2008). Serum trace elements in tube-fed neurological dysphagia patients correlate with nutritional indices but do not correlate with trace element intakes: case of patients receiving enough trace elements intake. Clin Nutr.

[10] Ruangritchankul S, Sumananusorn C, Sirivarasai J, Monsuwan W, Sritara P (2023). Association between dietary zinc intake, serum zinc level and multiple comorbidities in older adults. Nutrients.

[11] Sözüer DT, Barutçu UB, Karakoç Y, Yalçin E, Onen S (1995). The effects of antiepileptic drugs on serum zinc and copper levels in children. J Basic Clin Physiol Pharmacol.

[12] Eissa MA, Abdulghani KO, Nada MA, Elkhawas HM, Shouman AE, Ahmed NS (2020). Serum zinc and copper levels in a sample of Egyptian epileptic children. Egypt J Neurol Psychiatry Neurosurg.

[13] Verrotti A, Lattanzi S, Brigo F, Zaccara G (2020). Pharmacodynamic interactions of antiepileptic drugs: From bench to clinical practice. Epilepsy Behav.

